# An *In silico* analysis on the phosphorylation dependent structural and thermal stability of thermophilic proteins

**DOI:** 10.3389/fchem.2026.1796912

**Published:** 2026-05-06

**Authors:** Sermarajan Arunachalam, Ramachandran Gnanasekaran

**Affiliations:** Vellore Institute of Technology- Chennai, Chennai, India

**Keywords:** communication mapping, molecular dynamic simulations, phosphorylation, steered dynamics, thermophilic proteins, vibrational energy transfer

## Abstract

In the present study the effect of phosphorylation on the structural stability and energy transfer in two structurally homologous thermophilic proteins, 1QMP and 1DZ3 were investigated. In 1QMP one of the residues (*Asp55*) phosphorylated while 1DZ3 this modification is not observed. This modification has provided an ideal model to explore the influence of phosphorylation on thermostability of the protein. Molecular dynamics (MD) simulations were performed to assess the structural stability and flexibility of both proteins following the analyses of root-mean-square deviation and root-mean-square fluctuation (RMSF). Subsequent, vibrational energy transfer calculations were followed to characterize pathways of intramolecular energy propagation and also to evaluate the effect of phosphorylation *via* residue level communication within the protein matrix. The key residues and interaction networks involved in energy transport were visualized through communication maps, provided insights into influence of phosphorylation on intramolecular energy flow and also on the overall structural stability. Furthermore, steered molecular dynamics simulation were carried out to reveal the unfolding mechanism and thermal resistance of the proteins under the influence of applied force. The results have revealed that the 1QMP possess higher structural rigidity, enhanced energy transport efficiency, and greater resistance to unfolding as compared to 1DZ3. Overall, phosphorylation at *Asp55* strengthens vibrational energy transfer pathways and contributes to the enhanced thermostability of thermophilic proteins.

## Graphical Abstract

**Figure:**
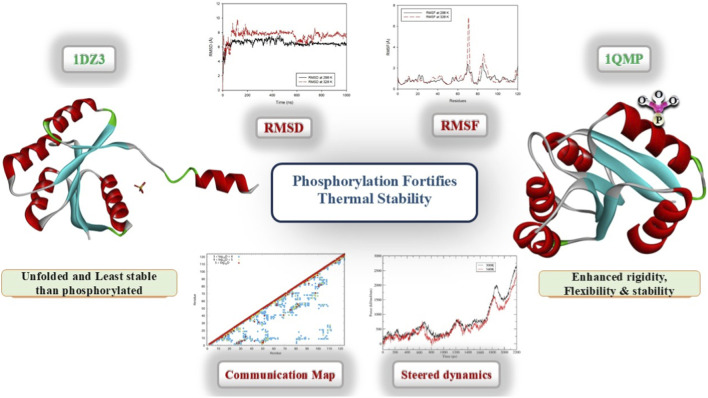

## Introduction

Thermophilic proteins are able to maintain their structure integrity and biological function even at very high temperatures. Understanding the stability of these proteins is important for enzyme design and industrial applications (Sen and Sarkar, 2022; Borhani and Arab, 2023). Several intrinsic factors are responsible for the thermal stability of thermophilic proteins. These include a higher proportion of charged and hydrophobic residues, an increased number of salt bridges, hydrogen bonds, and stronger core packing interactions that minimize the structural flexibility of the proteins (Wu et al., 2023; Zhou and Pang, 2018; Qing et al., 2022). Enhanced electrostatic interactions, improved secondary structure organization, and efficient vibrational energy diffusivity (VED) also play major roles in maintaining structural stability at elevated temperature (Cho and Rhee, 2021; De et al., 2024; Sterpone and Melchionna, 2012). Together, these molecular features enable thermophilic proteins to preserve their native conformation and resist thermal denaturation under extreme environmental conditions.

Moreover, Protein’s biological functions depend on their capacity to transfer vibrational energy effectively (Hunt, 2024; Löffler et al., 2022). The vibrational energy transport approach provides valuable information about energy transmit through the protein matrix and identifying key residues involved in micro-stability and flexibility, which are essential for proper function (Löffler et al., 2022; Ishizaki and Fleming, 2021; Yamato et al., 2022; Buchenberg et al., 2016; Bhat et al., 2024; Gnanasekaran and Pandey, 2024). Earlier studies such as by those Mithra D et al., emphasized comparative *in silico* analysis of thermophilic and mesophilic proteases revealed that thermophilic variants contain more charged and polar residues, extensive salt-bridge networks, including a novel cyclic salt bridge and a higher number of metal ion binding sites. Additionally, long-range aromatic-aromatic interactions contribute to enhanced rigidity. These structural features collectively explain the superior thermal stability of thermophilic proteins and provide valuable insights for protein engineering applications. (Mitra and Das Mohapatra, 2021).

In addition to these factors, post-translational modifications (PTMs) such as phosphorylation (Nishiwak et al., 2025), glycosylation (Tian et al., 2021), and ubiquitination (Sun and Zhang, 2022), play an important role in protein structure, dynamic, function and stability (Li et al., 2023). Among these, phosphorylation is one of the most widespread and functionally significant mechanisms of signal transduction and metabolic regulation in all living cells. It modulates key cellular processes by reversibly adding phosphate groups to specific amino acid residues, thereby influencing enzyme activity and conformational stability (Mitra and Das Mohapatra, 2021). Various amino acid including serine, threonine, tyrosine, histidine, and aspartate can act as phosphate acceptors (Barske and Hagemann, 2025; Yang et al., 2023). In particular, histidine and aspartate phosphorylation form the core of the two-component regulatory systems in bacteria, which govern adaptive processes such as chemotaxis, virulence, antibiotic resistance, and sporulation. In these systems, a sensor kinase transfers a phosphoryl group from ATP to a histidine residue, which is then communicated to an aspartate residue on the corresponding response regulator. This phosphorylation triggers conformational changes that activate downstream effector domains, leading to specific physiological responses (Lewis et al., 1999; Gupta and Gupta, 2021; Shaw et al., 2022). Aberrant phosphorylation pathways have also been implicated in various diseases, including cancer. Therefore, understanding the role of phosphorylation is essential for elucidating protein stability, dynamics, and thermoadaptation, making it a critical focus in structural biology and protein engineering (Mitra and Das Mohapatra, 2021).

Post-translational modifications (PTMs) play a crucial for regulating protein function and intermolecular interaction. In a broader biological context, PTMs are known to modulate immune checkpoint proteins and influence the efficacy of immunotherapies, including immune checkpoint inhibitors (ICIs). Therefore, understanding how PTMs affect protein flexibility and stability provides valuable insights into cellular signaling pathways, as well as the structural resilience and thermal adaptability of thermophilic proteins (Jia et al., 2025; Hu et al., 2025).

Recent proteomic studies have demonstrated that phosphorylation can modify protein thermostability in a site-specific manner (Smith et al., 2021). Large-scale thermal profiling experiments has shown that phosphorylated sites may either stabilize or destabilize proteins depending on their local structural environment. For instance, phosphosites located at protein–protein interfaces can reduce stability, whereas those buried within stable domains often enhance it (Smith et al., 2021).

In our earlier studies, we provided microscopic details into protein flexibility and conformational stability through the vibrational energy diffusivity methodology. VED calculations helped to identify the flexible and rigid residues responsible for the thermal denaturation at different temperatures. The results revealed that thermostabilization is primarily a consequence of reduced flexibility, whereas higher VED values correspond to larger fluctuations that disrupt organized structures and diminish functional activity. Moreover, residues involved in salt-bridge formation were found to be comparatively less dynamic, contributing to enhanced thermal stability. These observations were further correlated with the thermodynamic parameters of the system such as enthalpy, entropy, and heat capacity demonstrating that the VED approach is well-suited for investigating protein stability at the molecular level (Arunachalam and Gnanasekaran, 2024).

In this study, we performed a computational investigation of two structurally similar thermophilic proteins (1QMP and 1DZ3) to understand how phosphorylation and other intrinsic factors influence heat flow, vibrational energy transfer, and overall thermostability. Both proteins share a high degree of sequence similarity, but the key structural difference is the presence of a phosphorylated aspartate residue at position 55 (*Asp55*) in 1QMP, whereas 1DZ3 lacks this modification. This distinction provides an ideal model system for probing the influence of phosphorylation on protein stability and energy dynamics at the atomic level. Molecular dynamics simulations were performed for both proteins, and structural stability and flexibility were assessed through root-mean-square deviation (RMSD) and root-mean-square fluctuation (RMSF) analyses at two different temperatures. Based on VED calculations, a communication map was constructed to visualize energy-transport pathways and identify vibrationally active residues contributing to protein micro-stability. Additionally, steered molecular dynamics (SMD) simulations were carried out to explore mechanical unfolding and thermal stability under external force. Numerous applications of steered molecular dynamics (SMD) have been reported to evaluate the binding affinity in processes such as protein folding and protein–ligand interactions, following the general principle that the larger the rupture force, the higher the binding affinity (Chwastyk et al., 2014) (Nguyen et al., 2020) (Truong and Li, 2018) (Gunnoo et al., 2018) (Evans, 1979) (Zhao et al., 2017). SMD has also been widely applied in bioinformatics and rational drug design, providing molecular-level insights into binding mechanisms and energy landscapes (Do et al., 2018; Li, 2017; Iida and Tomoshi, 2022).

### Computational methods

Crystal structures of the response regulator protein Spo0A (PDB IDs: 1QMP and 1DZ3) (Lewis et al., 1999; Lewis et al., 2000), were obtained from the Protein Data Bank (Lewis et al., 2000; Lewis et al., 1999)1QMP (phosphorylated) has a resolution of 1.65 Å with 128 residues, while 1DZ3 (domain-swapped) has a resolution of 2.0 Å with 124 residues. The protein structures were visualized and preprocessed using BIOVIA Discovery Studio Visualizer 2021, where water molecules, ligands, and additional chains were removed prior to simulation setup (Arunachalam et al., 2025). Molecular dynamics (MD) simulations were performed in triplicate with different seed velocities using the Desmond module implemented in the Schrödinger Maestro Suite to ensure statistical robustness and enhanced conformational sampling, thereby enabling reliable evaluation of the structural stability and flexibility of both thermophilic proteins. Each protein system was placed in an orthorhombic simulation box and solvated using the TIP3P water model (Arunachalam et al., 2025) through the System Builder tool. Sodium chloride (NaCl) was added to achieve a physiological ionic strength of 0.15 M, and counterions were included to neutralize the system. The simulations were conducted under NPT ensemble conditions employing the OPLS4 force field at a temperature of 300 K and pressure of 1.013 bar, with a total simulation time of 500 ns. The root-mean-square deviation (RMSD) and root-mean-square fluctuation (RMSF) analyses were performed to assess global structural stability and residue-level flexibility throughout the trajectories.

To explore VED and communication networks within the proteins, additional analyses were carried out using GROMACS 2023.3 (Diaf et al., 2024) The phosphorylated protein (1QMP) was modelled with a covalently bound PO_3_^2−^ group at *Asp55*. All necessary force-field parameters for the phosphoryl moiety were developed and optimized to ensure accurate representation of its charge distribution and bonding characteristics. Both systems were then subjected to energy minimization using the steepest descent algorithm for 10,000 steps (Fliege and Fux, 2000). Subsequently, the proteins, including crystallographic water molecules, were placed in a cubic simulation box (62 Å^3^) and solvated using the TIP3P water model that had been pre-equilibrated.

Long-range electrostatic forces were computed using the particle-mesh Ewald (PME) approach, and periodic boundary conditions were applied with standard cutoff values (Petersen, 1995). The system was maintained at 1 atm pressure and 300 K throughout the simulation. After equilibration, structural snapshots were collected at 50, 100, 150, 200, and 300 ps. Only the water molecules solvating the protein were included, resulting in a system of roughly 4,000 atoms. These configurations were initially refined using the conjugate-gradient energy minimization protocol for approximately 50,000 iterations (Watowich et al., 1988), reaching an energy convergence threshold of 1 × 10^−6^ kcal/mol. They were then subjected to an extended 500,000-step minimization using the L-BFGS algorithm (Najafabadi et al., 2017), achieving a tighter convergence criterion of 1 × 10^−7^ kcal/mol before performing normal mode analysis (NMA). The resulting eigenmodes, eigenvectors, and Hessian matrices were used to generate frequency-resolved communication maps, elucidating residue–residue vibrational coupling and energy propagation pathways within the protein matrix. The detailed protocol for constructing communication maps follows our earlier methodology (Arunachalam and Gnanasekaran, 2024) and the original formulation (Leitner, 2009).

Further to probe the mechanical responses of homologous protein pairs under external force, steered molecular dynamics (SMD) simulations were performed in triplicates to ensure statistical reliability. The equilibrated structures obtained from the GROMACS production runs were used as starting geometries for the pulling simulations. A harmonic potential with a force constant of 1,500 kJ mol^−1^ nm^−2^ was applied between two defined groups -chain A (representing the main body of the protein) and chain B (the terminal residue used as the pulling handle) using the umbrella pulling scheme, with the center-of-mass (COM) distance along the z-axis as the reaction coordinate. The pulling was carried out at a constant rate of 0.01 nm ps^−1^ using a harmonic spring potential, allowing controlled elongation of the protein complex. The system was simulated under NPT ensemble conditions at 300 K and 340 K and 1 bar, employing the Nose–Hoover thermostat (Weldon and Wang, 2024) the Berendsen barostat (Yang et al., 2024; Jones et al., 2022). A time step of 2 fs was used, and all bond lengths involving hydrogen atoms were constrained using the LINCS algorithm. Long-range electrostatic interactions were treated with the Particle Mesh Ewald (PME) method (Chen et al., 2021), with a real-space cutoff of 1.4 nm. Trajectories and force profiles were recorded every 1 ps to capture the unfolding pathway and quantify the corresponding rupture forces. The resulting trajectories were analysed using GROMACS utilities, and structural changes were visualized using VMD (Jones et al., 2022) to assess secondary structure deformation and overall unfolding behaviour during the pulling process.

## Results and discussion

### Molecular dynamics simulations

**FIGURE 1:**
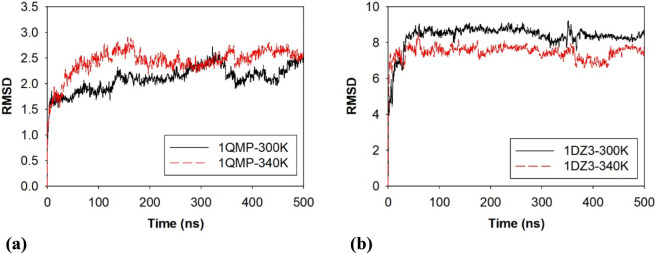
RMS deviation of **(a)** 1QMP and **(b)** 1DZ3 at 300K and 340K.

To investigate the dynamic behaviour of 1QMP and 1DZ3, molecular dynamic simulations were carried out. The RMSD was evaluated at different temperatures to follow the structural stability of 1QMP and 1DZ3 and also to quantify the extent of backbone conformational changes during the entire simulation period at different temperatures. The RMSD evaluations of 1QMP were performed for 100 ns at different temperatures between 288 K and 368 K to understand the microscopic behaviour of the proteins (Supplementary Figure S1). While the RMSD trajectories for the 1QMP and 1DZ3 proteins at two different temperatures (300 and 340 K) were performed for 500 ns and the same are shown in Figure 1. The initial variations in the RMSD plots were ignored, as they represent the equilibration phase where the proteins adjust from their starting structures to a stable form by relaxing steric clashes and rearranging solvent molecules.

Subsequently, the RMSD results obtained at two different temperatures revealed no significant structural deviations in 1QMP throughout the simulation period, indicated its high structural stability. In contrast, the 1DZ3 protein displayed slight variations in RMSD upon varying the temperature conditions, suggesting minor temperature-induced conformational changes in the protein backbone. The average RMSD values for the 1QMP proteins at 300K and 340K are 1.77 ± 0.889 Å and 2.81 ± 1.134 Å and 1DZ3 is 8.36 ± 2.959 Å and 7.46 ± 2.496 Å.

**FIGURE 2:**
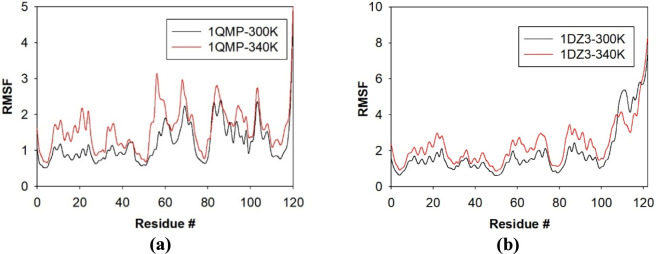
RMS fluctuation of **(a)** 1QMP and **(b)** 1DZ3 at 300K and 340K.

Root Mean Square Fluctuations (RMSF) provided valuable insights into the dynamic stability and flexibility of proteins during molecular dynamics simulations. In this study, the positional fluctuations of all amino acid residues in 1QMP and 1DZ3 were analysed at two different temperatures (Figure 2). The trajectory for 1QMP at different temperatures ranging between 288 and 368 K is shown in Supplementary Figure S2. (Arunachalam et al., 2025). Most of the residues exhibiting noticeable fluctuations were located within or near flexible regions of the protein structures. The 1QMP protein showed minimal residue fluctuations, whereas 1DZ3 exhibited slightly higher fluctuations under identical conditions. The average RMSF values for the 1QMP at 300K and 340K are 2.25 ± 1.2 Å and 2.28 ± 1.62 Å respectively while for 1DZ3 it is 3.38 ± 1.88 Å and 3.71 ± 2.32 Å.

In 1QMP, residues *Ser23, Pro26, Met58, Phe83* and *Leu105* displayed increased mobility, while in 1DZ3, residues *Glu20, Ser23, Met58, Gly63, Gly73, Lys93, Glu97, Glu111* and *Ile117* exhibited higher fluctuations (as compared to 1QMP) as the temperature increased from 300 K to 340 K. The residue *Ser23* and *Met58* showed increased fluctuations in both the proteins system.

### Communication mapping

**FIGURE 3:**
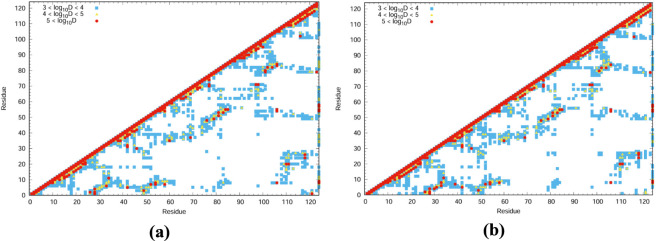
Communication mapping of 1QMP at **(a)** 300K and **(b)** 340K.

**FIGURE 4:**
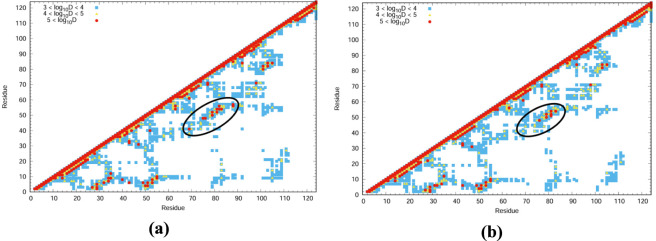
Communication mapping of 1DZ3 at **(a)** 300K and **(b)** 340K.

Communication maps were generated to visualize the vibrational energy transfer between residue pairs within the protein systems (Arunachalam and Gnanasekaran, 2024). Residues exhibiting higher vibrational energy distribution (VED) values are highlighted using a color-coded scheme to distinguish their relative contributions. High-energy residues were represented in red, followed by orange, and blue for gradually lower energy levels. These maps (Figures 3, 4) clearly identify the residues acting as major hubs for intramolecular energy transfer, provided insights into the communication pathways responsible for efficient energy flow. The VED values were derived from thermally averaged low-frequency modes, considering the density of ten modes centred at 50, 100, 150, 200, 300, and 400 cm^−1^. The resulting trends are consistent with those known to reflect enhanced conformational entropy in the folded state and the increased heat capacity characteristic of thermophilic systems (Gnanasekaran et al., 2011).

**FIGURE 5:**
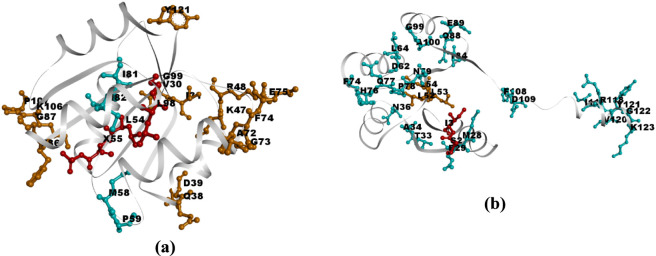
High vibrational active residues obtained from the Communication map for **(a)** 1QMP and **(b)** 1DZ3.

In Figures 3, 4, the communication maps of 1QMP and 1DZ3 are presented at two different temperatures, 300 K and 340 K. The comparison between these thermophilic proteins revealed clear differences in their residue communication patterns at different temperatures. At 300 K, both systems exhibited lower communication activity, indicating limited vibrational energy transfer between residues. When the temperature increases to 340 K, the 1QMP system showed a pronounced enhancement in residue communication, whereas 1DZ3 remains comparatively less active. This Suggested that 1QMP possesses a greater ability to transfer vibrational energy at elevated temperatures. The major structural distinction between these two proteins arises from the presence of a phosphoryl (PO_3_^2−^) group attached to the 55th aspartate residue in 1QMP. This group strengthens the inter-residue interactions and facilitates more effective energy transfer throughout the protein structure. For example, In 1QMP, residue *Asp55* exhibited a clear vibrational energy transfer pathway with residue *Phe86*. Furthermore, residue *Asp55* showed comparatively high vibrational energy diffusivity, as evidenced by the results demonstrated in Figure 5a. This suggested that residue *Asp55* may play a significant role in mediating energy redistribution within the protein network. Meanwhile, In the 1DZ3 structure, residues *Asp55* and *Ile56* exhibited distinct vibrational energy transfer interactions with *Gln77* and *Pro78* as observed from the communication mapping. Additionally, these residues are characterized by relatively high vibrational energy diffusivity, as illustrated in Figure 5b, indicating their potential involvement in efficient energy propagation within the protein framework. Overall, the results suggested that phosphorylation enhances both the thermal stability and communication efficiency of 1QMP compared to 1DZ3.

From these maps, residues within the high diffusivity range were identified and visualized in the corresponding protein structures (Figures 5a,b). These figures highlight the key residues involved in energy transport in both protein systems. Here as well, to illustrate variations in energy transfer, residues were color-coded according to their diffusivity values: high-energy residues are shown in red, followed by orange, and blue, representing progressively lower energy ranges. Red indicates values exceeding 10,000,000 Å/ps, orange represents 9,000,000–10,000,000 Å/ps, and blue corresponds to 8,000,000–9,000,000 Å/ps.

**FIGURE 6:**
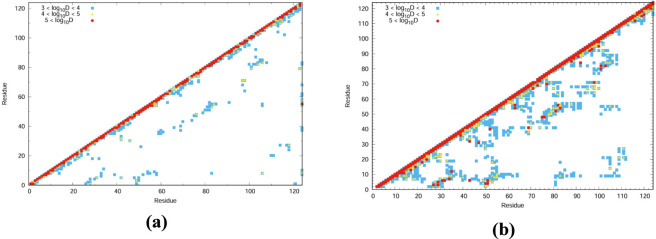
Communication map: Energy difference (T340K-T300K) for the **(a)** 1QMP and **(b)** 1DZ3.

**FIGURE 7:**
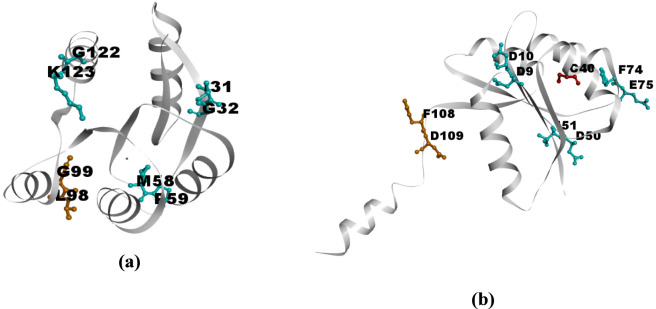
Temperature difference plot (340–300K) for **(a)** 1QMP and **(b)** 1DZ3.

To examine the residues involved in structural reorganization and stabilization upon increasing the temperature from 300 K to 340 K, difference plots were generated by comparing the VED values between the two temperatures. The corresponding communication plots for 1QMP and 1DZ3 are shown in Figures 6a,b, and the associated residues mapped onto the protein structures are presented in Figures 7a,b.

These residues exhibited significant vibrational activity and were found to play a critical role in energy redistribution as well as in the stabilization or destabilization of the thermophilic protein systems. The vibrationally active residues displayed high flexibility and were considered sources of entropy that contribute to protein stabilization or destabilization by locally disrupting the protein matrix. The contribution of salt bridges to system stability was also examined. The vibrational energy diffusion between salt-bridged residues was found to lie within a substantial range (10,000–1,000,000 Å/ps), though not within the range typically associated with stabilization.

From Figure 7a, it is observed that the phosphoryl group (PO_3_^2−^) and its covalently bonded aspartic acid residue are vibrationally active and played a major role in the signalling pathways within the system. In addition, residues such as *Leu98* and *Gly99* also exhibit strong vibrational activity, as indicated by their orange coloration in Figure 7a. These results suggested that the presence of the PO_3_^2−^ group facilitates vibrational energy transport along specific residue pathways, while other residues such as *Leu98* and *Gly99*, although not directly part of the main energy channel, also contribute to energy transfer due to their flexible conformations and higher energy states. This flexibility enables the protein to dynamically adjust its structure in response to temperature changes.

These delocalization of vibrational energy diffusivity arise from non-covalent interactions such as hydrogen bonding, π–π stacking, and salt bridges, which facilitate efficient energy transfer across the molecular framework. It is also important to note that not all vibrationally active communications necessarily lead to protein denaturation; rather, their effect depends on how energy is exchanged among the residue network. In the present case, as the protein structure remains stable upon increasing the temperature to 340 K, it can be inferred that the collective communication among multiple vibrationally active residues enables the system to overcome local free-energy barriers, thereby reinforcing structural stability at elevated temperatures. However, beyond a certain temperature threshold, these weak non-covalent interactions may begin to weaken and break, leading to the detachment of structural elements and, ultimately, protein denaturation.

A relatively high specific heat capacity indicated that a system can absorb a large amount of heat with only a small increase in temperature. The above results demonstrated that the phosphorylated 1QMP exhibited a lower specific heat capacity compared to the unphosphorylated 1DZ3 protein. This observation is consistent with the enhanced structural rigidity and reduced conformational fluctuations of 1QMP at elevated temperatures. The lower heat capacity can be attributed to limited atomic fluctuations arising from stronger hydrogen bonding, stable non-covalent interactions, and restricted residue mobility. These features collectively indicated that phosphorylation enhances the thermal stability of 1QMP by minimizing energy dissipation through structural fluctuations.

### Steered dynamics simulation

**FIGURE 8:**
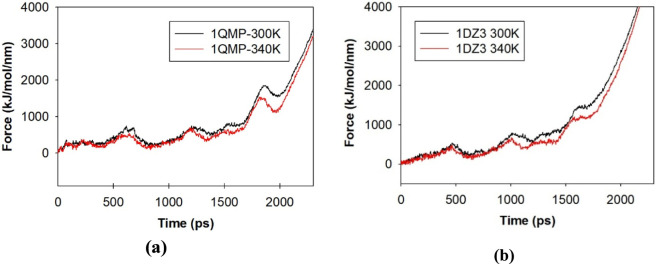
Steered dynamics plot for **(a)** 1QMP and **(b)** 1DZ3 at 300K and 340K.

**FIGURE 9:**
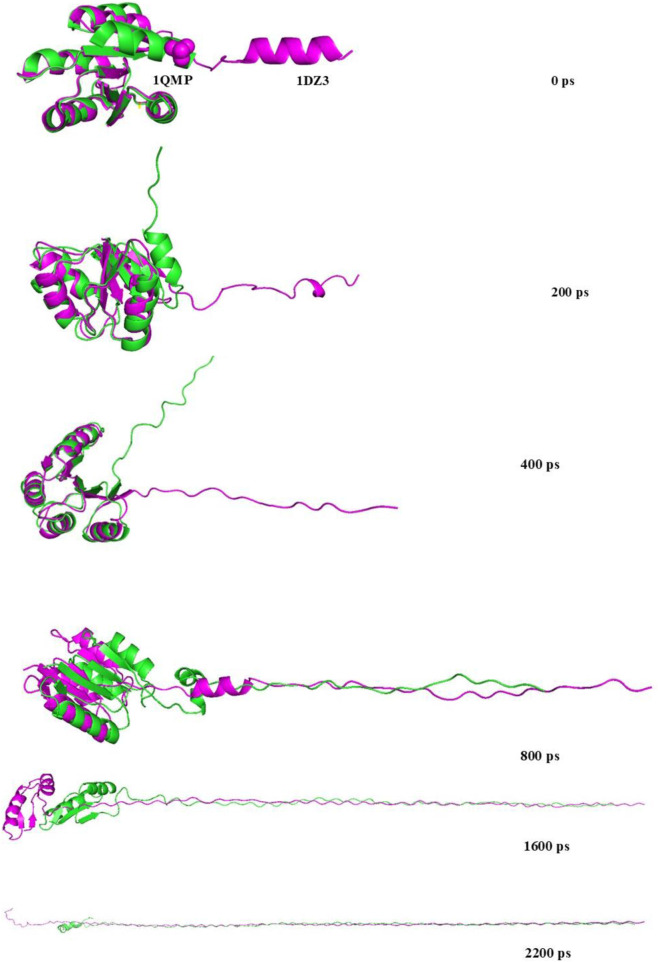
Denaturation at different time intervals 0ps, 200 ps, 400 ps, 800 ps, 1600 ps and 2200 ps.

The steered molecular dynamics (SMD) simulations were performed to evaluate the mechanical unfolding behavior of 1QMP and 1DZ3 proteins under identical pulling conditions at 300 K and 340 K. The resulting force - time plot (Figure 8) revealed distinct differences in the mechanical responses of the two proteins, providing insight into their relative structural stabilities (Chavoshpour and Farzi, 2025; Isralewitz et al., 2001). Both proteins exhibited a gradual increase in pulling force with time, indicating resistance to unfolding; however, the force required to unfold 1QMP remained consistently higher than that of 1DZ3 across the entire trajectory. Figure 9 clearly showed the denaturation trajectories occurred at different timescales such as 200 ps, 400 ps, 800 ps, 1,600 ps and 2,200 ps respectively.

In 1QMP, the force increased gradually and reached a maximum of approximately 2,800 kJ mol^−1^ nm^−1^ at 300 K, whereas 1DZ3 attained only around 2,600 kJ mol^−1^ nm^−1^ under the same conditions. The higher force in 1QMP indicated stronger internal interactions such as hydrogen bonds, salt bridges, and hydrophobic contacts, which collectively contribute to its greater mechanical stability. Upon increasing the temperature to 340 K, both proteins showed a reduction in the required unfolding force due to enhanced molecular motion and flexibility. However, 1QMP still required higher forces than 1DZ3, confirming its superior thermal flexibility. The major resistance to unfolding in 1QMP originates from the residues 79–128 including C-terminal α-helix, which is stabilized by nine hydrogen bonds shown in the Supplementary Data S1. This region exhibited strong rigidity, reflected by distinct force peaks in the range of 1,500–2,000 kJ mol^−1^ nm^−1^, suggesting that the helix remains closely packed and intact even under significant mechanical stress. In contrast, the corresponding region in 1DZ3 contains only two hydrogen bonds, resulting in a more flexible and less stable helix that unfolds at lower forces (∼1,200–1,500 kJ mol^−1^ nm^−1^). Consequently, the weaker hydrogen-bond network in 1DZ3 leads to an earlier phase of denaturation.

Furthermore, in 1QMP, the phosphorylated aspartate residue (*Asp55*) within residues 33–50 forms a strong hydrogen-bonding with aspartate 62 that stabilizes the protein core, effectively preventing unfolding until the applied force exceeds ∼700 kJ mol^−1^ nm^−1^. This stabilizing interaction is absent in 1DZ3, leading to earlier disruption of the central region and reduced overall stability. At the N-terminal segment (residues 1–49), 1QMP maintains two conventional hydrogen bonds up to forces beyond 2,500 kJ mol^−1^ nm^−1^, preserving structural integrity throughout the pulling process. In contrast, 1DZ3 loses these interactions at around 1,800 kJ mol^−1^ nm^−1^, which contributes to premature structural denaturation. The force time plot of 1QMP displayed broader and more defined peaks, indicated the presence of stable intermediate states during stretching. These intermediates reflected stronger intramolecular interactions that resist unfolding transitions. Conversely, 1DZ3 showed more frequent force fluctuations and an earlier force decay, consistent with weaker structural rigidity and faster unfolding under mechanical load.

Overall, the SMD results demonstrated that 1QMP possesses greater mechanical and thermal stability than 1DZ3. The higher unfolding forces and smoother transition patterns observed for 1QMP reflected its tightly packed and rigid architecture, dominated by an extensive hydrogen-bonding network and stable α-helical organization. In contrast, the lower unfolding forces and irregular force profile of 1DZ3 suggested a less compact, more flexible structure, explaining its lower resistance to both mechanical and thermal stress.

## Conclusion

Comprehensive molecular dynamics (MD) and steered molecular dynamics (SMD) simulations were carried out to understand the microscopic behaviour, structural stability, and energy communication characteristics of two thermophilic proteins, 1QMP and 1DZ3, under different temperature conditions. The RMSD and RMSF analyses revealed that 1QMP maintained a compact and stable conformation throughout the simulations, showing only minor backbone fluctuations, whereas 1DZ3 exhibited higher deviations and increased flexibility at elevated temperatures. These observations indicated that 1QMP possesses superior conformational stability and resistance to temperature-induced unfolding. Communication mapping provided further evidence of the robust residue–residue interactions and efficient vibrational energy transfer within 1QMP. The presence of a phosphoryl (PO_3_^2−^) group covalently attached to *Asp55* in 1QMP played a critical role in strengthening intramolecular communication networks and enhancing energy delocalization across the protein framework. In contrast, 1DZ3, lacking this modification, demonstrated weaker communication pathways and reduced thermal resilience. Steered molecular dynamics simulations further validated these findings by showing that 1QMP required a higher unfolding force compared to 1DZ3, reflecting stronger intramolecular interactions such as hydrogen bonds, salt bridges, and hydrophobic contacts. Even under elevated temperatures, 1QMP maintained structural integrity, while 1DZ3 unfolded more readily. Overall, the results highlight that phosphorylation significantly contributes to improved thermostability, mechanical strength, and energy transfer efficiency in thermophilic proteins. The insights gained from this study deepen our understanding of molecular mechanisms underlying protein stabilization and can guide the rational design of thermostable enzymes for industrial and biotechnological applications.

## Data Availability

The raw data supporting the conclusions of this article will be made available by the authors, without undue reservation.
