# Supplementary material for: An In silico analysis on the phosphorylation dependent structural and thermal stability of thermophilic proteins
Source: Front Chem. 2026 May 6;14:1796912. doi: 10.3389/fchem.2026.1796912 (PMC13187877; doi:10.3389/fchem.2026.1796912)
Supplement: Supplementary file 1 [file Supplementaryfile1.docx]

**An *In Silico* Analysis on the Phosphorylation Dependent Structural and Thermal Stability of Thermophilic Proteins**

Sermarajan Arunachalam, Balamurali MM and Ramachandran Gnanasekaran*

Vellore Institute of Technology- Chennai 600127

**Supplementary Information**

**Supplementary Information 1**

**Steered Molecular dynamics simulations**

**List of Hydrogen bonding existed in 1QMP at different time frames**

**Hydrogen bonding existed upto 400 ps**

*Ile81-Ala100*

*Ile 81-Ser101*

*Ile81-Tyr102*

*Ile81-Phe103*

*Leu83-Phe103*

*Leu83-Ile104*

*Thr84-Leu105*

*Ala85- Leu105*

*Leu83- Leu105*

**Hydrogen bonding existed upto 800 ps**

*Asp55-Asp62*

**Hydrogen bonding existed upto 1800 ps**

*Lys3-Glu28*

**Hydrogen bonding existed upto 2200 ps**

*Ser1-Asp26*

*Ile2-Met27*

**List of Hydrogen bonding existed in 1DZ3 at different time frames**

**Hydrogen bonding existed upto 400 ps**

*Ile 81-Ser101*

*Ile81-Tyr102*

**Hydrogen bonding existed upto 1800 ps**

*Ser1-Asp26*

*Ile2-Met27*

Figure S1. RMSD trajectories of 1QMP at different temperatures.

Figure S2. RMSF trajectories of 1QMP at different temperatures.
